# Trends and regional variations in the administrative prevalence of attention-deficit/hyperactivity disorder among children and adolescents in Germany

**DOI:** 10.1038/s41598-018-35048-5

**Published:** 2018-11-19

**Authors:** M. K. Akmatov, A. Steffen, J. Holstiege, R. Hering, M. Schulz, J. Bätzing

**Affiliations:** 1 Department of Regional Health Care Analysis and Health Care Atlas, Central Research Institute of Ambulatory Health Care in Germany (ZI), Berlin, Germany; 2 Department of Health Services Research and Risk Structure, Central Research Institute of Ambulatory Health Care in Germany (ZI), Berlin, Germany

## Abstract

There is a controversy regarding temporal trends in prevalence of attention-deficit/hyperactivity disorder (ADHD). Using nationwide claims data containing data for approximately six million statutory health insured children we aimed to examine a) trends of ADHD administrative prevalence during 2009–2016; b) regional variations in prevalence, and c) factors associated with an increased chance of ADHD diagnosis. The ICD-10 code ‘F90-hyperkinetic disorder’ was used to define an ADHD case. Global and Local Moran’s I tests were used to examine the spatial autocorrelation and k-means-cluster analysis to examine the course of ADHD prevalence in administrative districts over years. Two-level logistic regression was applied to examine individual- and district-level factors associated with ADHD diagnosis. The administrative prevalence of ADHD was 4.33% (95% CI: 4.31–4.34%). We observed pronounced small-area differences on the district level with prevalences ranging between 1.6% and 9.7%. There was evidence of strong spatial autocorrelation (Global Moran’s I: 0.46, p < 0.0001). The k-means-method identified six clusters of different size; all with a stagnating trend in the prevalence over the observation period of eight years. On the district level, a lower proportion of foreign citizens, and a higher density of paediatric psychiatrists and paediatricians were positively associated with ADHD with a more pronounced effect in urban districts.

## Introduction

Attention-deficit/hyperactivity disorder (ADHD) is a common neurodevelopmental disorder primarily affecting children and adolescents (from here on referred to as children). There is probably no other mental disorder more controversially discussed both by the public and scientific community. First, prevalence varies substantially between and within countries. ADHD appears to be a public health issue in western countries with the highest prevalence estimates reported in the US and lowest in African and Asian countries^1^. Globally, the reported ADHD prevalence varies between 1% and 7%^2^. Regional variations within countries were also reported, *e.g*. in Germany^3^, the UK^4^ and the US^5^. It is argued that regional variations can be explained by sociodemographic factors, health care access or availability of medical specialists^3^, but evidence from empirical studies is lacking. Second, there is a controversy regarding temporal trends of the disease frequency. A few studies reported about rising trends of disease frequency (reviewed in^6^). In Germany, an increase in prevalence was reported by two studies^3,7^; *e.g*. from 2.5% in 2005 to 4.2% in 2015^3^ and from 5.0% in 2009 to 6.1% in 2014^7^, although some studies showed no increase in recent years. The question arising in this context is whether the prevalence increase over the last years is true or whether it is just the result of the heightened attention from the public, media and scientific community. Using nationwide claims data containing data for approximately six million children we examined a) temporal trends of ADHD administrative prevalence estimates during 2009 to 2016; b) regional variations in ADHD prevalence, including small-area variations, and c) sociodemographic factors on the individual and district level associated with an increased chance of ADHD diagnosis.

## Results

### ADHD administrative prevalence and regional differences

**Figure 1:**
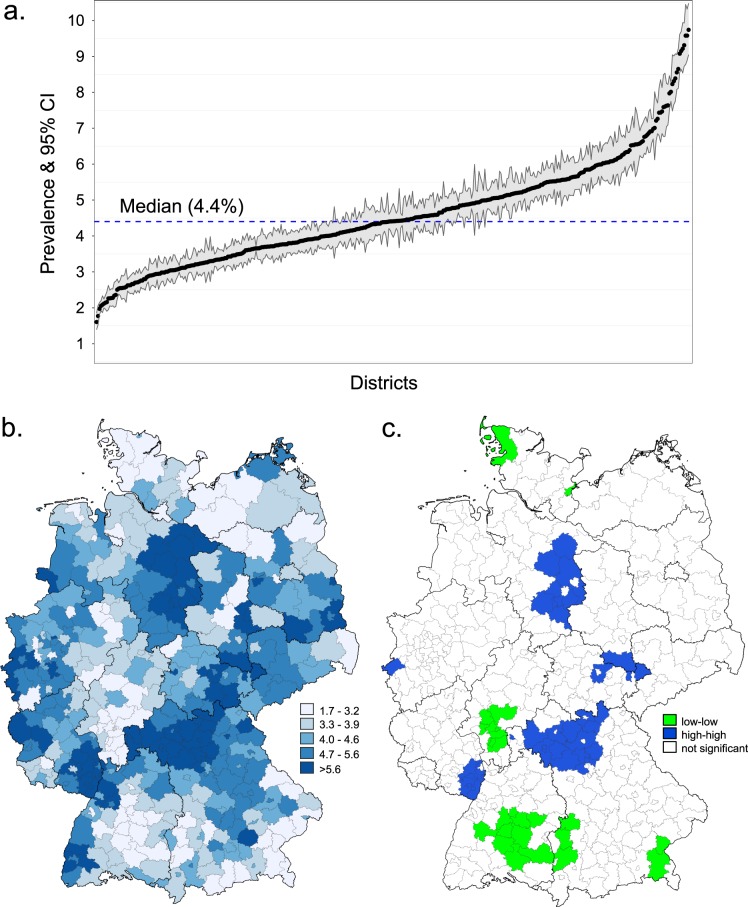
Administrative prevalence of ADHD among children and adolescents by districts, Germany in 2016. (**a**) Caterpillar plot of ADHD prevalences in 402 administrative districts with 95% confidence intervals (sorted by the magnitude of the prevalence). 95% confidence intervals were calculated according to Wilson. (**b**) Cartographic representation of ADHD prevalences by district. (**c**) Districts with significant spatial effects estimated with Local Moran’s I^19^.

The dataset consisted of approximately six million children residing in 402 districts (Table 1). This made up around 82% of all children in Germany (Table 1, column 4). The distribution of the study population according to sex, age and federal state was in good agreement with that of the general German population (Table 1). Approx. 260,000 children lived in Germany with ADHD in the year 2016. This corresponds to an administrative prevalence of 4.33% (95% confidence intervals, CI: 4.31–4.34%). We observed pronounced small-area differences with prevalences ranging between 1.6% and 9.7% (Fig. 1a,b). The Global Moran’s I statistic showed a strong evidence of spatial autocorrelation which decreased almost linearly over the years (Global Moran’s I: 0.58 (2009), 0.56 (2011), 0.53 (2013), 0.49 (2015) and 0.46 (2016)), but remained significant at the level of p < 0.0001 in all years. Applying Local Moran’s I statistics we identified two groups of clusters with low-low and high-high prevalences (Fig. 1c). Districts with significantly similar high prevalence estimates were observed in the Northern part of Bavaria, the Southern part of Rhineland-Palatinate, the East of Lower Saxony, and a cluster including districts of Thuringia, Saxony-Anhalt and Saxony. Districts with low-low prevalence estimates were found in the Southern and Northern parts of Germany. The number of districts with spatially autocorrelated prevalences decreased from 45 in 2009 to 38 in 2016. In support of these results, the median odds ratio from the null model was 1.36 (95% credible interval: 1.33–1.39), *i.e*. children living in districts with a lower probability of getting an ADHD diagnosis would have a 1.36-times higher chance to be diagnosed with ADHD if he/she would move to a district with a higher probability of getting an ADHD diagnosis.

**Table 1:** Comparison of selected characteristics of the study population with the general German population by sex, age group, and Federal States. *Data from the Federal Statistical Office for the year 2016.

Characteristics	Study population		General German population*	
	*n* (*N* = *6,007,414*)	*Percent*	*n* (*N* = *7,292,122*)	*Percent*
Sex				
Male	3,085,299	51.4	3,750,712	51.4
Female	2,922,115	48.6	3,541,410	48.6
Age groups				
5–6 years	1,249,953	20.8	1,435,639	19.7
7–8 years	1,203,030	20.0	1,448,518	19.9
9–10 years	1,177,430	19.6	1,445,268	19.8
11–12 years	1,179,998	19.6	1,466,514	20.1
13–14 years	1,197,003	19.9	1,496,183	20.5
German federal states				
Baden-Württemberg	783,699	13.0	1,005,201	13.8
Bavaria	910,163	15.2	1,139,837	15.6
Berlin	256,383	4.3	305,426	4.2
Brandenburg	177,860	3.0	213,354	2.9
Bremen	50,213	0.8	57,242	0.8
Hamburg	130,048	2.2	155,861	2.1
Hesse	455,454	7.6	562,139	7.7
Mecklenburg-Western Pomerania	117,972	2.0	133,171	1.8
Lower Saxony	604,374	10.1	721,521	9.9
North Rhine-Westphalia	1,350,041	22.5	1,614,938	22.1
Rhineland-Palatinate	279,402	4.7	354,210	4.9
Saarland	66,177	1.1	78,819	1.1
Saxony	302,777	5.0	342,006	4.7
Saxony-Anhalt	156,058	2.6	175,225	2.4
Schleswig-Holstein	211,710	3.5	257,978	3.5
Thuringia	155,083	2.6	175,194	2.4

### Temporal development of ADHD administrative prevalence

**Figure 2:**
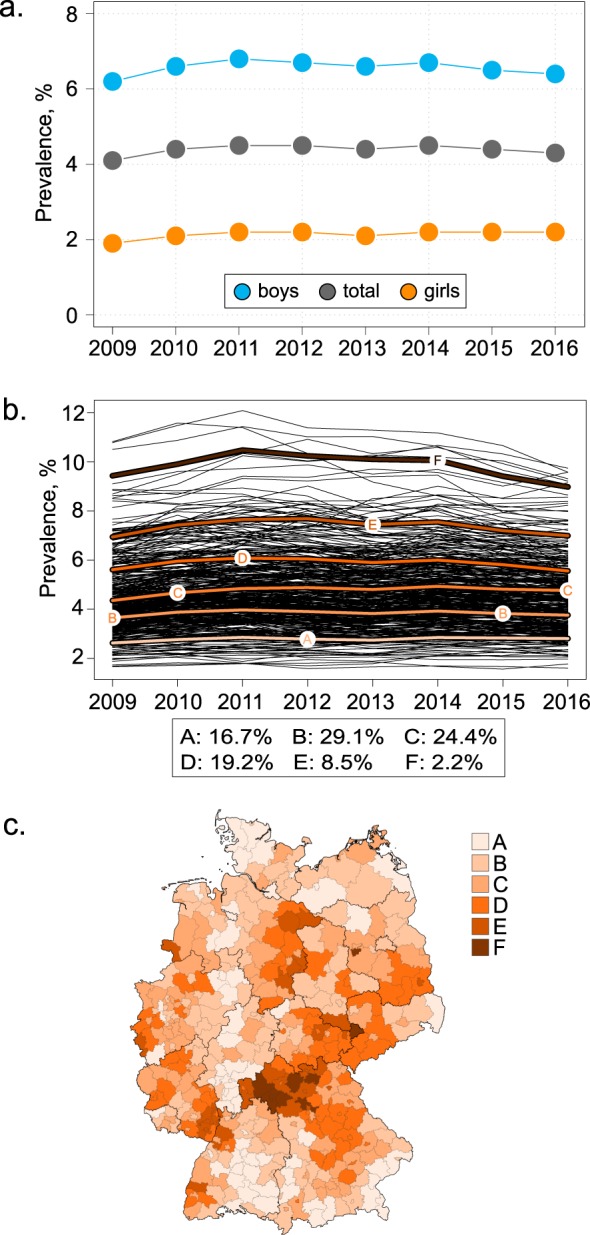
Administrative prevalence of ADHD among children and adolescents in Germany. (**a**) Prevalence in the years 2009 to 2016: total and by sex and (**b**) by administrative districts. Black lines in panel (b) represent prevalence courses over the years in 402 districts; coloured lines with the letter symbols represent the identified trajectories. K-means-cluster analysis for longitudinal data was used to identify the trajectories^20^. (**c**) Cartographic representation of the trajectories from the panel (b).

The administrative prevalence of ADHD did not show an increasing trend (Fig. 2a). The k-means-method resulted in six trajectories of different size (Fig. 2b). All trajectories displayed a stagnating trend in the prevalence, however, they differed in prevalence levels. In one cluster (trajectory F) there was a slightly increasing prevalence up to the year 2011 and decline afterwards. As expected, cartographic representation of the trajectories revealed a similar picture as seen in Fig. 1b. Of note, seven of the nine districts in the trajectory F (with the highest prevalence) were observed in the northern part of Bavaria.

### Factors associated with ADHD diagnosis

Boys were three-times more likely to be diagnosed with ADHD than girls (Table 2). The chance of ADHD diagnosis increased with advancing age and was highest among 11-year-old children as compared to 5-year-old children (adjusted odds ratio: 6.21; 95% CI: 6.04–6.38) and decreased slightly thereafter. On the district level, a lower proportion of foreigners, higher density of both paediatric psychiatrists and paediatricians were positively associated with ADHD diagnosis (Table 2). These effects were more pronounced in urban districts (Table 2, fifth column) and were not significant in other districts.

**Table 2:** Factors associated with an ADHD diagnosis (results of two-level multivariable logistic regression analysis with approximately six million children and adolescents (level 1) residing in 402 districts (level 2). *The models adjusted for all variables in the table. The MOR and PCV were calculated using the formulas proposed by Merlo *et al*.^18^.

Variables	Total sample (*n = 402 districts*)	Rural areas with a low population density (*n = 96 districts*)	Rural areas with population concentrations (*n = 103 districts*)	Urban districts (*n = 136 districts*)	Big urban municipalities (*n = 66 districts*)
**Fixed effects at the individual level**	**AOR & 95% CI**	**AOR & 95% CI**	**AOR & 95% CI**	**AOR & 95% CI**	**AOR & 95% CI**
Sex: boys vs. girls	**3.10 (3.07–3.13)**	**3.09 (3.02–3.16)**	**3.15 (3.08–3.22)**	**3.11 (3.06–3.15)**	**3.06 (3.01–3.12)**
Age					
6 vs. 5 years	**1.70 (1.65–1.76)**	**1.65 (1.53–1.78)**	**1.67 (1.55–1.80)**	**1.75 (1.66–1.84)**	**1.69 (1.59–1.80)**
7 vs. 5 years	**3.04 (2.95–3.13)**	**2.73 (2.54–2.93)**	**2.86 (2.66–3.06)**	**3.23 (3.09–3.39)**	**3.08 (2.91–3.26)**
8 vs. 5 years	**4.61 (4.48–4.74)**	**3.97 (3.71–4.25)**	**4.41 (4.13–4.72)**	**4.92 (4.70–5.15)**	**4.71 (4.46–4.97)**
9 vs. 5 years	**5.67 (5.52–5.83)**	**4.84 (4.53–5.18)**	**5.61 (5.26–5.99)**	**6.03 (5.77–6.31)**	**5.75 (5.46–6.06)**
10 vs. 5 years	**6.15 (5.98–6.32)**	**5.35 (5.01–5.72)**	**6.00 (5.62–6.40)**	**6.54 (6.26–6.84)**	**6.22 (5.91–6.56)**
11 vs. 5 years	**6.21 (6.04–6.38)**	**5.32 (4.98–5.69)**	**6.15 (5.76–6.57)**	**6.64 (6.35–6.94)**	**6.22 (5.90–6.56)**
12 vs. 5 years	**6.08 (5.91–6.24)**	**5.26 (4.92–5.61)**	**6.18 (5.79–6.59)**	**6.45 (6.17–6.75)**	**6.01 (5.70–6.34)**
13 vs. 5 years	**5.86 (5.70–6.02)**	**5.08 (4.75–5.42)**	**5.91 (5.53–6.31)**	**6.23 (5.96–6.51)**	**5.80 (5.51–6.13)**
14 vs. 5 years	**5.32 (5.18–5.47)**	**4.55 (4.26–4.87)**	**5.54 (5.19–5.91)**	**5.57 (5.32–5.83)**	**5.34 (5.06–5.63)**
**Fixed effects at the district level**					
Place of residence					
rural areas with a low population density	**1.14 (1.00–1.30)**	—	—	—	—
rural areas with population concentrations	1.07 (0.94–1.21)	—	—	—	—
urban districts	1.12 (0.99–1.25)	—	—	—	—
big urban municipalities	reference				
Socioeconomic deprivation index					
lowest deprivation	reference	reference	reference	reference	reference
low deprivation	0.97 (0.88–1.08)	1.47 (0.74–2.91)	0.95 (0.76–1.19)	0.91 (0.79–1.05)	1.10 (0.87–1.40)
medium deprivation	1.01 (0.91–1.13)	1.48 (0.76–2.89)	0.98 (0.79–1.23)	0.95 (0.82–1.11)	0.95 (0.74–1.21)
high deprivation	**1.12 (1.00–1.26)**	1.62 (0.83–3.15)	1.14 (0.91–1.42)	1.09 (0.93–1.28)	1.03 (0.79–1.34)
highest deprivation	1.00 (0.90–1.12)	1.42 (0.72–2.79)	1.11 (0.88–1.41)	0.95 (0.78–1.17)	0.89 (0.72–1.11)
Proportion of individuals with a foreign citizenship					
lowest quintile	**1.29 (1.10–1.51)**	0.78 (0.54–1.14)	1.71 (0.89–3.31)	**1.55 (1.20–2.00)**	1.32 (0.95–1.84)
low quintile	**1.24 (1.07–1.44)**	0.84 (0.56–1.25)	1.68 (0.88–3.21)	**1.42 (1.12–1.80)**	0.96 (0.72–1.28)
middle quintile	**1.22 (1.05–1.40)**	0.80 (0.53–1.20)	1.63 (0.86–3.09)	**1.43 (1.13–1.81)**	1.04 (0.82–1.33)
high quintile	1.10 (0.96–1.26)	—	1.52 (0.77–2.99)	1.16 (0.94–1.45)	1.14 (0.94–1.39)
highest quintile	reference	reference	reference	reference	reference
Density of paediatric psychiatrists (per 100,000 citizens)					
lowest quintile (<0.61)	reference	reference	reference	reference	reference
low quintile (0.61–0.90)	1.02 (0.93–1.12)	1.12 (0.96–1.32)	1.04 (0.88–1.23)	0.92 (0.78–1.07)	0.93 (0.71–1.22)
middle quintile (0.91–1.20)	1.23 (0.91–1.13)	0.98 (0.79–1.21)	**1.32 (1.13–1.54)**	**1.24 (1.09–1.41)**	1.16 (0.90–1.50)
high quintile (1.21–1.80)	**1.22 (1.00–1.26)**	1.08 (0.83–1.40)	1.17 (0.92–1.50)	**1.19 (1.01–1.39)**	1.21 (0.86–1.69)
highest quintile (>1.80)	1.22 (0.90–1.12)	1.22 (0.99–1.50)	1.20 (0.99–1.45)	**1.40 (1.15–1.71)**	1.03 (0.79–1.36)
Density of paediatricians (per 100,000 citizens)					
lowest quintile (<5.40)	reference	reference	reference	reference	reference
low quintile (5.40–6.20)	0.94 (0.86–1.03)	1.02 (0.85–1.22)	0.86 (0.73–1.01)	0.93 (0.78–1.07)	0.83 (0.43–1.61)
middle quintile (6.21–7.10)	0.96 (0.87–1.06)	1.08 (0.89–1.32)	0.82 (0.68–0.98)	**1.24 (1.09–1.41)**	0.97 (0.48–2.00)
high quintile (7.11–8.50)	1.06 (0.95–1.19)	1.17 (0.85–1.63)	1.03 (0.83–1.28)	**1.19 (1.01–1.39)**	0.77 (0.40–1.47)
highest quintile (>8.50)	**1.12 (1.01–1.24)**	1.06 (0.82–1.37)	1.08 (0.88–1.33)	**1.22 (1.01–1.47)**	0.90 (0.47–1.71)
**Random effects**					
Variance (SE) from the empty model	0.107 (0.0077)	0.098 (0.0147)	0.099 (1.0143)	0.111 (0.0137)	0.108 (0.0192)
Variance (SE) from the final model	0.092 (0.0067)	0.089 (0.0133)	0.081 (0.0116)	0.070 (0.0087)	0.080 (0.0143)
MOR (95% Crl) from the empty model	1.36 (1.33–1.39)	1.35 (1.29–1.40)	1.35 (1.29–1.40)	1.37 (1.32–1.42)	1.37 (1.29–1.44)
MOR (95% Crl) from the final model	1.33 (1.30–1.36)	1.33 (1.27–1.38)	1.31 (1.26–1.36)	1.29 (1.24–1.32)	1.31 (1.24–1.37)
PCV	−14%	−10%	−19%	−37%	−25%

AOR, adjusted odds ratio; CI, confidence intervals; Crl, credible interval; MOR, median odds ratio; PCV, proportional change in variance; SE, standard error.

## Discussion

Using nationwide claims data of approximately six million children in Germany we examined regional differences in ADHD administrative prevalence, its temporal trends and factors on the individual and district level associated with a higher chance of ADHD diagnosis. In contrast to other studies we observed no increase in administrative prevalence of ADHD in Germany over the years 2011 to 2016. This trend was relatively stable across sex and administrative districts. Most of the districts displayed a similar temporal pattern. Globally, findings regarding temporal trends in ADHD prevalence are controversial. For example, two US studies reported a considerable increase of parent-reported ADHD diagnosis made by a health professional from 2003 to 2011^8,9^. However, our finding is in agreement with results from other international studies. For example, in a metaanalysis of 135 cross-sectional studies conducted at different timepoints Polanczyk *et al*. did not observe an increase in ADHD prevalence over the last 30 years^2^. They concluded that variability in ADHD prevalence estimates is predominantly explained by methodological differences of the studies^2^. We used claims data of several years and applied standardized methodology (*e.g*. in terms of a study population and a case definition). Controlling for such methodological differences apparently resulted in the absence of prevalence increase.

We found substantial regional variations in ADHD prevalence. First, children living in rural areas were more likely to be diagnosed with ADHD than their counterparts from urban areas. However, after controlling the model for the proportion of foreign citizens on the district level – which was taken as proxy for individuals with migration background – this effect was strongly diminished. Apparently, children from migrant families may be underdiagnosed either due to a poor access to health care facilities and/or cultural differences in perception of ADHD-related symptoms. The latter is confirmed by systematic reviews which reported higher prevalence estimates in industrialized countries such as the US as compared to African and Asian countries^1^. Second, we observed small-area differences in ADHD prevalence which decreased over the years but were still substantial in 2016. Multilevel regression analysis showed that the individual probability of being diagnosed with ADHD was also dependent on children’s residence. The corresponding MOR from the empty model was 1.36, meaning that children were 1.36-times more likely to be diagnosed with ADHD if they would move from a district with a lower ADHD prevalence to a district with a higher prevalence. We examined which factors might explain regional differences by controlling for individual- and district-level variables in a two-level regression model. The associations of ADHD diagnosis and sex and age were similar to those shown in other studies. On the district level we also found the availability of paediatric psychiatrists and paediatricians significantly associated with being diagnosed with ADHD. It can be thus concluded that the regional variation in ADHD prevalence can partly be explained by both, under- and over-diagnosing. The association of a higher risk of ADHD diagnosis and a lower proportion of migrants indicates to an underdiagnosing problem as mentioned above. On the other side, the positive association of ADHD diagnosis and the availability of psychiatrists may be a result of over-diagnosing. The latter has already been observed in several studies. For example, a US study identified that the risk of diagnosis with autism increased among children who moved into neighbourhoods with a better access to health care^10^. Overall, the examined variables on the district level only explained part of the regional variation. Other factors such as smoking, alcohol consumption or stress during pregnancy, or exposure to lead or polychlorinated biphenyls, which may vary geographically, may explain further regional variations in ADHD prevalence.

### Strengths and limitations

First, we used claims data containing information on approximately 87% of all individuals in Germany. When comparing our study population with the general German population we found minor differences in terms of sex, age and federal state distribution. Our dataset contains no information about children insured privately (around 13% of the general population). Privately insured individuals tend to have a higher socioeconomic status than statutory insured individuals and may be less affected by ADHD^11^. We may thus slightly overestimate the prevalence regarding the total German population. Second, routinely collected claims data are secondary data collected for purposes other than *e.g*. prevalence estimation. It is well known that physicians may misdiagnose ADHD, although considerable efforts have been done in Germany to improve diagnostics and therapy of common mental disorders, including ADHD. One of them is introduction of the Psychotherapist Law in 1999 in Germany according to which psychological psychotherapists and psychotherapists specializing in children and adolescents were integrated into the ambulatory health care services. This resulted in the improvement of health care of patients with mental disorders. In addition, children are regularly examined by paediatricians from local public health services. Paediatricians work in close cooperation with nursery-school teachers and school teachers and in case of a suspicious ADHD a child is transferred to a specialist. In spite of these improvements, the risk of misdiagnosis cannot be ruled out definitively. For example, a study of Wolraich *et al*. showed that a substantial proportion of US paediatricians do not follow the clinical guidelines for diagnosis and treatment of children with ADHD^12^. In this study every fifth paediatrician does not use formal diagnostic criteria of ADHD and every third paediatrician does not use rating scales. Bruchmüller *et al*. examined the extent of under- and overdiagnosis of ADHD among paediatric psychotherapists in four German Federal States^13^. They observed that around 20% of children who fulfilled diagnostic criteria of ADHD did not receive an ADHD diagnosis, *i.e*. underdiagnosis. On the contrary, around 17% of children who do not fulfil the diagnostic criteria received falsely ADHD diagnosis, *i.e*. overdiagnosis. As a result, true prevalences may be under- or overestimated. To minimize the latter we applied a conservative definition of ADHD case. An alternative approach to estimate ADHD prevalence is a cross-sectional study design involving parents. However, relying solely on parental reports may be prone to bias (*e.g*. recall or reporting bias). Epidemiological studies should rather use standardized instruments, including structured interviews with parents and if possible with teachers and rating scales^14^. Third, due to the large sample size we were able to carry out robust small-area estimations. For example, the average sample size across all districts was 15,000 children. The absolute number of ADHD cases varied between 91 and 9373 across districts with an average of 650 cases, indicating that even in a district with the lowest number of ADHD cases (n = 91) we were able to provide robust estimates of ADHD prevalence.

One of the potential limitations of this study is the lack of individual-level data such as socioeconomic status or migration background. On the individual level the dataset contained only information on ADHD diagnosis, sex, age and place of residence. This was compensated by the application of multilevel modelling where we used aggregated data on the district level obtained from the literature. However, multilevel models can be prone to aggregation bias and thus need to be considered with caution.

## Conclusions

The administrative prevalence of ADHD among children in Germany did not show an increasing trend in recent years. However, there were considerable regional differences which remained significant after adjusting for demographic factors on the individual and socioeconomic factors on the district level. There is indication that migrant children may be underdiagnosed or less affected by this disorder. Further research is needed to investigate factors explaining remaining regional variations in ADHD prevalence in Germany.

## Methods

### Data and sample

We used ambulatory claims data of all statutory health insured children between 5 and 14 years of age in Germany in the years 2009 to 2016 (n = 6,007,414 in 2016). It is estimated that around 87% of the total German population is insured statutory; the remaining 13% of the population is insured privately. Claims data contain outpatient diagnoses of individuals who contacted physicians at least once per year. Diagnoses are coded according to the German modification of the International Classification of Diseases (ICD-10-GM). In addition, claims data contain information on individual’s sex and age and place of residence (the latter based on postal code). Because no other information on the individual level is available in claims data (*e.g*. sociodemographic status or migration background), we used publicly available data on the district level, including (a) socioeconomic deprivation index^15^, (b) density of paediatric psychiatrists, (c) density of paediatricians, *i.e*. number of physicians per 100,000 citizens^16^, and (d) proportion of individuals with a foreign citizenship^17^.

### Ascertainment of ADHD cases

We used the code ‘F90 – hyperkinetic disorder’ with all subcodes (*i.e*. F90.0, F90.1, F90.8 and F90.9). We considered a child having an ADHD if he/she had been diagnosed in at least two quarters of the corresponding year. This was done to reduce the proportion of false positive diagnoses as it is known that using coding the diagnosis only in one quarter of the year may overestimate the true prevalence.

### Statistical analysis

Initially, we calculated crude- and sex-specific prevalences of ADHD. Furthermore, we used the Global and Local Moran’s I test to examine spatial autocorrelation^18^ and the k-means-cluster-analysis for longitudinal data to examine the course of the prevalence in administrative districts over the years 2009–2016^19^ We then applied two-level logistic regression analysis with approximately six million children (level 1) residing in 402 districts (level 2) to examine individual- and district-related factors associated with ADHD diagnosis. We controlled the model for sex and age on the individual level and for: (a) socioeconomic deprivation index^15^, density of (b) paediatric psychiatrists and (c) paediatricians, *i.e*. number of physicians per 100,000 citizens^16^, (d) proportion of individuals with a foreign citizenship^17^, and (e) children’s place of residence with four categories (rural areas with a low population density, rural areas with population concentrations, urban districts and big urban municipalities) on the district level. Initially, we examined whether there was variation in ADHD diagnosis across districts; this was done in a model without predictors (*i.e*. null model). We calculated median odds ratio (MOR) as an indicator of unexplained community heterogeneity^18^. Then, we included all above mentioned variables and calculated the MOR again to examine whether the variables explained part of the variance in ADHD prevalence across the districts. Analyses were performed with STATA, version 15 (StataCorp LLC, College Station, Texas, USA) and the R Foundation for Statistical Computing, version 3.3.3. The R packages “spdep” and “KmL” were used to examine spatial autocorrelation and prevalence trajectories, accordingly.

### Ethics approval

The use of claims data for scientific research is regulated by the Code of Social Law (SGB X) in Germany. According to this law, an ethical approval and informed consent are not required as these are routinely collected pseudonymized data.

## Data Availability

The dataset analysed during the current study is available from the corresponding author on reasonable request.
